# Widespread Nanoparticle-Assay Interference: Implications for Nanotoxicity Testing

**DOI:** 10.1371/journal.pone.0090650

**Published:** 2014-03-11

**Authors:** Kimberly J. Ong, Tyson J. MacCormack, Rhett J. Clark, James D. Ede, Van A. Ortega, Lindsey C. Felix, Michael K. M. Dang, Guibin Ma, Hicham Fenniri, Jonathan G. C. Veinot, Greg G. Goss

**Affiliations:** 1 Department of Biological Sciences, University of Alberta, Edmonton, Alberta, Canada; 2 Department of Chemistry and Biochemistry, Mount Allison University, Sackville, New Brunswick, Canada; 3 Department of Chemistry, University of Alberta, Edmonton, Alberta, Canada; 4 Department of Biomedical Engineering, University of Alberta, Edmonton, Alberta, Canada; 5 National Institute of Nanotechnology, Edmonton, Alberta, Canada; National Institute of Health (NIH), United States of America

## Abstract

The evaluation of engineered nanomaterial safety has been hindered by conflicting reports demonstrating differential degrees of toxicity with the same nanoparticles. The unique properties of these materials increase the likelihood that they will interfere with analytical techniques, which may contribute to this phenomenon. We tested the potential for: 1) nanoparticle intrinsic fluorescence/absorbance, 2) interactions between nanoparticles and assay components, and 3) the effects of adding both nanoparticles and analytes to an assay, to interfere with the accurate assessment of toxicity. Silicon, cadmium selenide, titanium dioxide, and helical rosette nanotubes each affected at least one of the six assays tested, resulting in either substantial over- or under-estimations of toxicity. Simulation of realistic assay conditions revealed that interference could not be predicted solely by interactions between nanoparticles and assay components. Moreover, the nature and degree of interference cannot be predicted solely based on our current understanding of nanomaterial behaviour. A literature survey indicated that ca. 95% of papers from 2010 using biochemical techniques to assess nanotoxicity did not account for potential interference of nanoparticles, and this number had not substantially improved in 2012. We provide guidance on avoiding and/or controlling for such interference to improve the accuracy of nanotoxicity assessments.

## Introduction

As nanomaterial (NM) production and use continues to become more prevalent, consistent and accurate NM toxicity testing is crucial for the ability to properly regulate these materials. Many conflicting reports on the potential toxicity of NMs have made it difficult to predict their biological effects [1], [2]. One of the primary issues afflicting consistent toxicity testing may be the use of biochemical assays that can be affected by NMs themselves, leading to data artefacts and subsequent incongruent estimations of toxicity. Such inconsistent and/or inaccurate data will make it difficult for regulators to establish guidelines and procedures for NM production and use, ultimately hindering our ability to predict how NMs will affect organisms in the environment.

Due to the unique physicochemical properties and increased reactivity of nanoparticles (NPs), there is a high potential for these materials to interfere with spectrophotometric and spectrofluorometric assays. Commonly used tests such as the lactate dehydrogenase (LDH) cytotoxicity assay, alamar blue, and tetrazolium based assays (*e.g.* MTS and MTT) are frequently reported to be affected by a range of different NPs [3]–[6]. A large number of *in vivo* and *in vitro* nanotoxicology experiments include these or similar assays which are designed to quickly and efficiently assess toxicity. Many of these protocols rely on multi-step biochemical reactions resulting in changes in absorbance or fluorescence, which are then quantified to provide information on physiological or biochemical endpoints. A comprehensive review of assays used for nanotoxicity testing is beyond the scope of this discussion and can be found elsewhere [7]–[9], but most contain dyes or proteins with significant potential to interact with NPs.

NPs can bind to proteins [10]–[15] and dyes [16] and alter their structure and/or function, and it is probable that this process is occurring in common toxicity assays. The presence of NPs in assays may adversely influence these reactions and cause significant changes in enzyme activity [10]–[12], [15], fluorescence, and/or the absorbance characteristics of indicator molecules [17], [18]. Carbon-based NMs have been shown to consistently affect a number of toxicity assays and are documented to bind to dyes [19], [20]. Carbonaceous NPs bind to alamar blue [19], coomassie blue [19], neutral red [19], [20], MTT dye [2], [4], [19], [20], and WST-1 dye [19], and can therefore interfere with assays using these indicators [21].

To assess the extent of NP interference with toxicity assays and to determine if effects are predictable based on the physicochemical properties of the NP, we performed a systematic investigation of the accuracy of commonly used toxicity assays. First we tested assay reliability with only NPs and assay components, and then tested several of the procedures under more realistic conditions (*i.e.* with cellular debris or protein present). Since a number of papers have already reported interference of assays by carbonaceous materials, we chose to investigate a broader suite of other commonly used NPs: silicon (Si), cadmium selenide (CdSe), zinc oxide (ZnO), titanium dioxide (TiO_2_) NPs, and helical rosette nanotubes (RNTs). An additional goal was to review existing nanotoxicology literature to assess whether appropriate assay controls were performed in response to the reporting of NP-assay interference over the past few years. In this regard, we also analyze a subset of peer-reviewed papers from both 2010 and 2012 to determine what proportion employ a colorimetric or fluorometric biochemical assay, and to evaluate whether there has been improvement in the performance and reporting of controls for these assays.

## Materials and Methods

### Nanoparticle synthesis and characterization

Si functionalized with undecanoic acid were synthesized and suspended in double distilled water (ddH_2_O) according to Clark *et al.* [22]. CdSe were functionalized with mercaptoundecanoic acid and prepared in ddH_2_O as described by Zhong *et al.* [23]. Polyacrylic acid-capped ZnO and TiO_2_ were provided by ViveNano (Toronto, Canada). RNTs, an organic self-assembling nanotube composed of repeating units of heteroaromatic bicyclic bases conjugated to lysine, were synthesized and suspended in ddH_2_O according to Fenniri *et al.* [24].

The shape and size of NPs were determined with transmission electron microscopy analysis (TEM). The hydrodynamic diameter and zeta (ζ) potential of NPs in ddH_2_O were assessed with a Malvern Instruments (Westborough, MA) Zetasizer Nano ZS equipped with a 633 nm laser. Disposable cuvettes were cleaned with filtered water immediately before use, filled with NP suspension, and then capped. Hydrodynamic diameter measurements were acquired in 173° backscattering mode and reported as the peak value of >99% intensity. All ζ-potential and hydrodynamic diameter measurements were reported as the mean of minimum three measurements plus or minus one standard deviation about the mean. Concentrations lower than 1 mg/L in all NPs resulted in erroneous readings on the Zetasizer, and thus were not reported. Large standard errors were observed in the RNTs. This is possibly the result of polydispersity in the sample or it may relate to the assumption of diameter calculations that the particles were spherical; the tubular shape of the RNTs likely renders the dynamic light scattering (DLS) measurements inaccurate for this particle. NP stock suspensions were sonicated for 30 s using a wand type sonicator before preparation of test solutions, and suspensions were vortexed for 30 s before initiating experiments.

### Optical measurements

To determine the intrinsic fluorescence and absorbance of each NP an absorbance and fluorescence spectrum were plotted. NPs were diluted to 100 mg/L in ddH_2_O and 2 mL were pipetted into a cuvette. An absorbance spectrum from 300–600 nm (Hewlett Packard 8452A diode array spectrophotometer) and a fluorescence spectrum (excitation 535 nm, Cary Eclipse photoluminescence spectrometer) were recorded.

### Assay treatments

All assay validations, with the exception of the LDH cytotoxicity assay, were performed with the same protocol. A standard curve was calculated for each 96-well microplate assay and microplates were only used if the standard curve had an R^2^>0.99. In each experiment we first determined if the presence of NPs would result in the production of fluorescence or colour in the absence of analytes. NPs in ddH_2_O were added to kit reagents and the assay was run according to manufacturer's instructions. In a second series of experiments we simulated a more realistic assay scenario by adding an analyte; 40 µg/mL bovine serum albumin (BSA) for the Bradford assay, 250 µg/mL BSA for the Bicinchoninic (BCA) assay, a lysed mixture of 12.5×10^6^ cells/mL for the LDH assay, or 250 U/mL catalase for the Amplex Red Catalase assay was added to the microplate wells in addition to the NPs and kit reagents. This manipulation was not possible with the MTS or alamar blue assay since they are dependent on cellular metabolic activity, which is difficult to replicate experimentally. If live cells were used as an analyte, it would be difficult to distinguish differences between assay interference and actual effects of nanoparticles on cells. Results are reported as ‘difference in reported protein/catalase activity/number of cells’, which was calculated by subtracting the actual value of protein/catalase activity/number of cells added to the microplate well from the value reported by the assay. For example, if an assay reported 130 µg/mL of BSA and 40 µg/mL BSA was actually added, then the ‘difference in reported protein’ would be 90 µg/mL.

### Protein quantification assays (BCA and Bradford Assays)

Protein quantification assays were performed using BCA (Pierce Biotechnology, Rockford, IL, USA) and Bradford methods (BioRad Laboratories, Hercules, CA, USA). Microplate assays for both methods were performed according to manufacturer's recommendations and absorbance was measured on a Molecular Devices (Sunnyvale, CA, USA) Spectramax microplate reader. Briefly, for the BCA assay, protein reduces Cu^2+^ to Cu^+^ under alkaline conditions, and this cupric ion binds 2 molecules of BCA dye, which absorbs at 562 nm and is sensitive from 20–2000 µg/mL protein. The Bradford assay is a more direct method for protein quantification; Coomassie Brilliant Blue G-250 dye binds to aromatic and basic amino acid residues in proteins and the resulting complex absorbs at 595 nm and is sensitive from 200 to 900 µg/mL protein. In both cases, standard curves were prepared using BSA in ddH_2_O. Test samples consisted of ddH_2_O containing only NPs (0.1, 1, or 10 mg/L final concentration) or ddH_2_O with NPs and BSA.

### MTS cell proliferation/viability assay

MTS [3-(4,5-dimethylthiazol-2-yl)-5-(3-carboxymethoxyphenyl)-2-(4-sulfophenyl)-2H-tetrazolium] is a tetrazolium compound that can be bioreduced by metabolically active cells to a soluble formazan product. The quantity of formazan produced is indicative of the number of viable cells in culture and can be determined colorimetrically by recording the change in absorbance at 490 nm. This assay was performed according to manufacturer's recommendations for the Cell Titer 96 Aqueous Non-Radioactive Cell Proliferation Assay Kit (Promega, WI, USA). Briefly, Complete Minimal Essential Media (Hyclone) supplemented with 10% heat inactivated fetal bovine serum (Hyclone) and 2 mM _L_-glutamine (Gibco), 100 U/mL penicillin (Gibco), and 100 µg/mL streptomycin (Gibco) and was dispensed into a 96-well plate. NPs were added to each well to achieve final concentrations of 1, 10 or 100 mg/L. The kit reagents were then prepared, added to the plate, and incubated for an additional 2 h (37°C, 5% CO_2_). Absorbance (490 nm) was recorded using a microplate reader (WALLAC 1420, PerkinElmer, MA, USA). A standard curve with a rat basophilic leukemia (RBL) cell line, RBL 2H3, was run in parallel. RBL cells (5, 10, 20, 40, and 80×10^3^ cells/well) were used to determine changes in reported cell number indicating NP interference.

### Alamar Blue cell viability assay

AlamarBlue Cell Viability Reagent (Invitrogen, DAL1025) is commonly used to assess cell health. Simply, the metabolic activity of cells converts soluble resazurin dye into fluorescent resorufin, and fluorescence (excitation 535 nm, emission 590 nm) of this dye was recorded. For the standard curve, RBL 2H3 cells were seeded from 0–60×10^3^ cells/well, and incubated for 2 h (37°C, 5% CO_2_), after which the assay was run according to manufacturer's instructions. Final well concentrations of 1, 10, and 100 mg/L of each NP were used with the exception of Si, which were only measured at 1 and 10 mg/L due to low NP stock concentration.

### Catalase assay

Catalase is an enzyme important in the reduction of potentially harmful hydrogen peroxide into water and oxygen. Catalase activity was assessed with Molecular Probes' Amplex Red Catalase Assay Kit (A22180) and the assay was performed according to manufacturer's instructions. Briefly, Amplex Red reagent reacts in a 1∶1 ratio with hydrogen peroxide in the presence of horseradish peroxidase to produce the fluorescent molecule resorufin. When catalase is active, it decreases the concentration of hydrogen peroxide and thus the amount of resorufin. Absorbance (560 nm) and fluorescence (excitation 535 nm, emission 595 nm) were recorded using a microplate reader (Wallac 1420, Perkin Elmer). A standard curve of 0–1000 U/mL catalase was used to extrapolate the catalase activity levels, (“reported catalase levels”) and 1, 10, and 100 mg/L NP were used with the exception of Si, which were tested only at 1 and 10 mg/L due to low stock concentration.

### LDH cytotoxicity assay

Many commercially available cytotoxicity assay kits measure the activity of the intracellular enzyme lactate dehydrogenase (LDH), which can be released to the extracellular media by damaged cells. We have previously shown that NPs can inhibit or even abolish the activity of purified LDH [6] but it is not clear if this phenomenon will occur in more complex sample mixtures. LDH activity was assessed in RBL cells. The culture media contained substantial LDH enzyme activity (data not shown), and as such cells were washed to remove excess media. A known quantity of cells was centrifuged at 450 g for 7 min, the supernatant was removed, and the cells were resuspended in phosphate buffered saline. This process was repeated 3 times. Cells were lysed using a wand type sonicator (model SLPe, Branson Ultrasonics, Danbury, CT, USA) to release the LDH enzyme. The lysed mixture was centrifuged briefly at 450 g to remove cell debris and the final mixture was subsequently assayed for LDH activity by following the oxidation of β-NADH to β-NAD at 340 nm in a 96-well spectrophotometer (SoftMax Pro, Molecular Devices, Sunnyvale, CA, USA).

The cuvette-based protocol, described previously by MacCormack *et al.* [6] was modified for a 96-well plate. A seven-point standard curve was generated for each plate that ranged from 0 to 20×10^6^ cells/mL and run in parallel with LDH-containing test samples. Test samples were added to wells at a cell concentration of 12.5×10^6^ cells/mL and exposed to either a NP treatment or vehicle (ddH_2_O) in triplicate, and immediately assayed for β-NADH oxidation. Background oxidation was determined in the absence of pyruvate and was negligible for each plate assayed. The number of cells reported for each sample was calculated using the linear equation of the standard curve. Due to low stock concentration, only 1 mg/L of Si, 1 and 10 mg/L of CdSe and RNT, and 1, 10, and 100 mg/L of TiO_2_ and ZnO were measured.

### Statistics

Protein, enzyme, or cell concentrations were established using absorbance or fluorescence values from treatment wells and calculated using the equation determined from the standard curve on the same plate. Values are reported as differences between this calculated concentration and the actual concentration of protein, enzyme, or cell added to the plate. All statistics were calculated using Prism 4 using one-way analyses of variance (ANOVA) followed by post-hoc Dunnett's test. Statistical significance was set at *p*<0.05. Each treatment was repeated in triplicate per plate and each experiment replicated 3–6 times. Data are reported as the mean ± SEM.

### Survey of nanotoxicological papers

A survey of current papers published in the area of nanotoxicology was performed to determine the percentage of studies running and reporting controls for colorimetric or fluorescent-based assays. On 28 September 2010, a search for “nanoparticle toxicity assay” in Google Scholar was performed and the top 200 papers from 2010 were reviewed. The number of papers using a colorimetric or fluorescent assay were recorded, and of those, the number performing the following controls: 1) Measurement of the intrinsic fluorescence and absorbance of the NPs; 2) Assessment of the interference of NPs with the assay components and dyes; 3) Assessment of the interference of NPs with the assay components and dyes together with an analyte. On 14 November 2012, this search and analysis was repeated with the top 200 search results, limited to 2012 to determine whether there was a change in the reporting of controls in the literature.

## Results and Discussion

### Nanoparticle characterization

**Figure 1:**
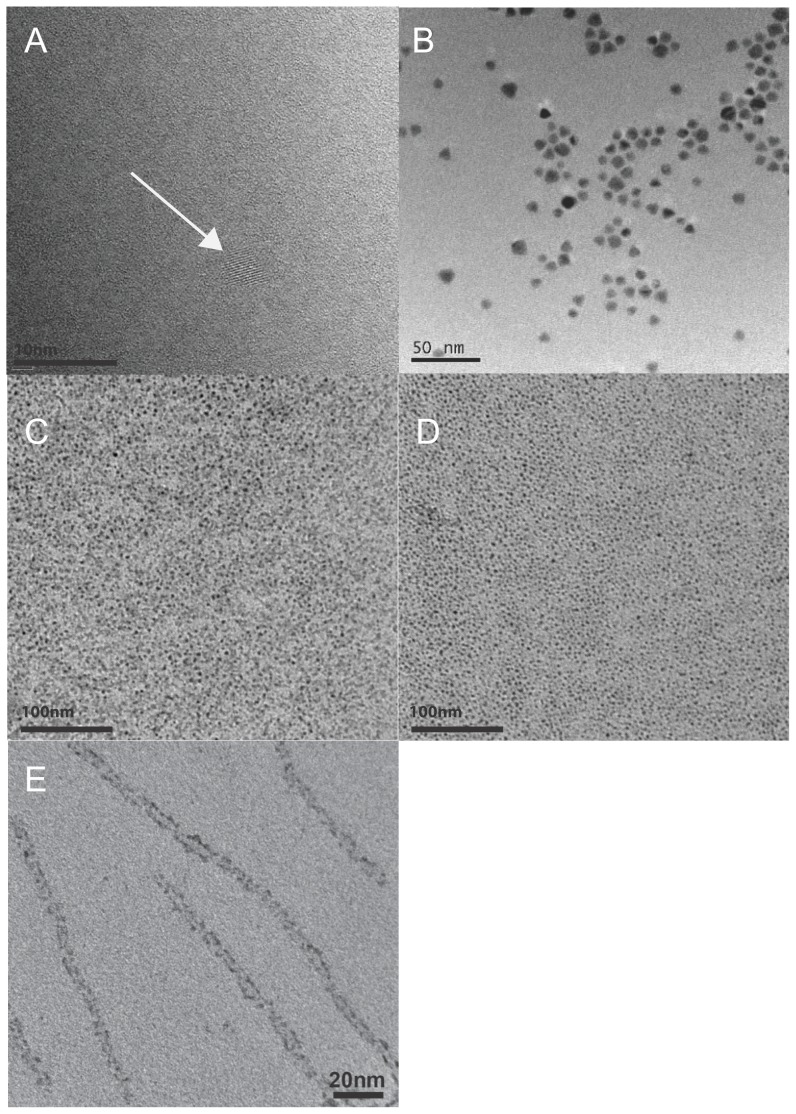
Transmission Electron Micrographs (TEM) of nanoparticles show that nanoparticle cores range from 3–9 nm. (A) High-resolution TEM of silicon nanoparticles functionalized with undecylenic acid, arrow points to Si crystalline structure; (B) Cadmium selenide nanoparticles functionalized with undecylenic acid; (C) Titanium dioxide nanoparticles functionalized with polyacrylic acid; (D) Zinc oxide nanoparticles functionalized with polyacrylic acid; (E) Helical rosette nanotubes functionalized with lysine.

To determine whether basic physicochemical NP characteristics could be used to predict interference with assays, hydrodynamic diameter, ζ-potential, and NP core size measurements were performed. Transmission electron micrographs, hydrodynamic size, and ζ-potential information for all NPs are provided in Table 1. Other physicochemical parameters of some of the NPs used here have also been reported previously (Si [25], RNTs [24]). NP core diameters ranged from 3–9 nm (Figure 1). TEM confirmed the spherical shape of each particle with the exception of tubular RNT (Figure 1). Hydrodynamic diameter of NPs in ddH_2_O ranged from 20–700 nm as determined by DLS, and NPs tended to agglomerate as concentration increased (Table 1). RNTs were too highly agglomerated (>1 µm) to allow for a reading at 100 mg/L on the DLS. NPs with similar functionalizations did not always follow the same pattern of hydrated diameter and agglomeration; for example, Si and CdSe NPs were of similar size at 1 mg/L (151±2 nm and 181±12 nm, respectively), but CdSe NPs agglomerated at 10 mg/L (240±8 nm) and 100 mg/L (703±13 nm) whereas the hydrodynamic diameter of Si NPs did not significantly change. The more labile binding of the mercaptoundecylenic acid functionalizations to CdSe NPs likely results in greater variations in hydrodynamic diameter at different concentrations than the covalently capped Si NPs. Steric stabilization by polymer coating of TiO_2_ and ZnO generally resulted in smaller agglomerations than CdSe and Si. At 100 mg/L, TiO_2_ and ZnO had similar hydrated diameters (150±1 nm and 153±12 nm, respectively), but varied at 1 mg/L (20±2 nm and 118±13 nm) and 10 mg/L (25±1 nm and 109±1 nm). CdSe NPs had the most negative ζ-potential (−52±2 mV) while other particles ranged from −26 to −52 mV, with the exception of RNTs, which were strongly positive (+77 mV) (Table 1). These variations in hydrated diameter and charge were predicted to alter potential interactions with charged dyes and proteins in the assays.

**Table 1:** Physicochemical characterization of nanoparticles.

	Functionalization	Source	Hydrodynamic diameter (nm)^a^			Zeta potential (mV)^b^	
			1 mg/L	10 mg/L	100 mg/L	10 mg/L	100 mg/L
**Si**	undecylenic acid	Veinot [24]	151±2	150±1	147±2	−28±3	−43±6
**CdSe**	undecylenic acid	Veinot	181±12	240±8	703±13	−47±0	−52±2
**TiO_2_**	polyacrylic acid	Vive Nano	118±13	109±1	150±1	−26±2	−31±1
**ZnO**	polyacrylic acid	Vive Nano	20±2	25±1	153±12	−30±5	−35±3
**RNT**	lysine	Fenniri [23]	344±200	544±334	N/A	77±4	73±4

a Hydrodynamic diameter as measured by DLS and reported as mean±standard error.

b Zeta potential as measured by Zetasizer and reported as mean±standard error.

### Optical measurements

**Figure 2:**
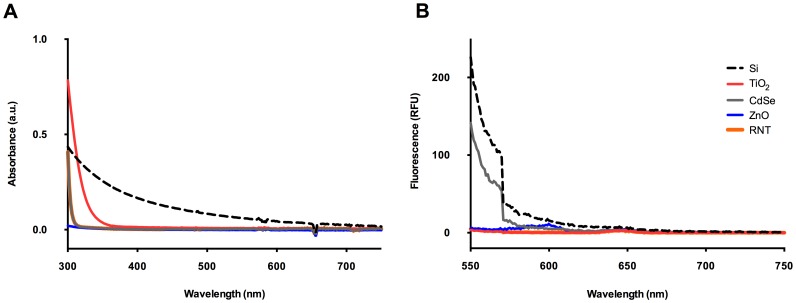
Spectroscopic measurement of optical characteristics of nanoparticles. (A) Absorbance of nanoparticles; (B) Fluorescence (RFU) of nanoparticles (excitation, 531 nm).

**Figure 3:**
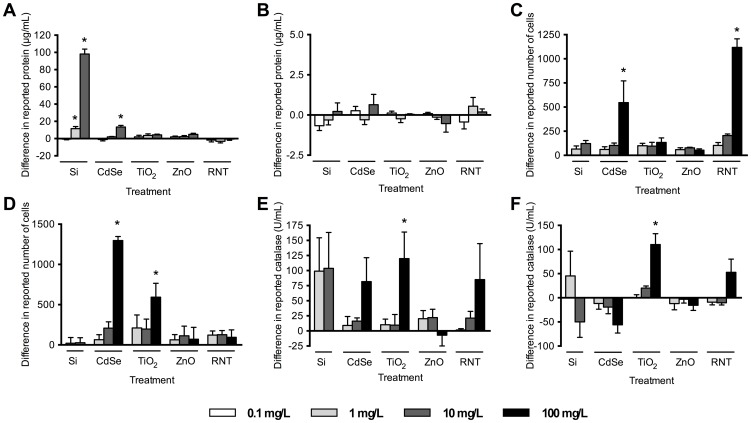
Assessment of nanoparticle interference with the assay components, without analyte. (A) BCA (bicinchoninic) protein assay; (B) Bradford protein assay, (C) MTS (3-(4,5-dimethylthiazol-2-yl)-5-(3-carboxymethoxyphenyl)-2-(4-sulfophenyl)-2H-tetrazolium) assay; (D) Alamar blue assay (excitation 531 nm, emission 595 nm); (E) Amplex red catalase assay (excitation 531 nm, emission 595 nm); (F) Amplex red catalase assay (emission 560 nm). * indicates significantly different than control (p<0.05, ANOVA followed by Dunnett's post-hoc comparison).

**Figure 4:**
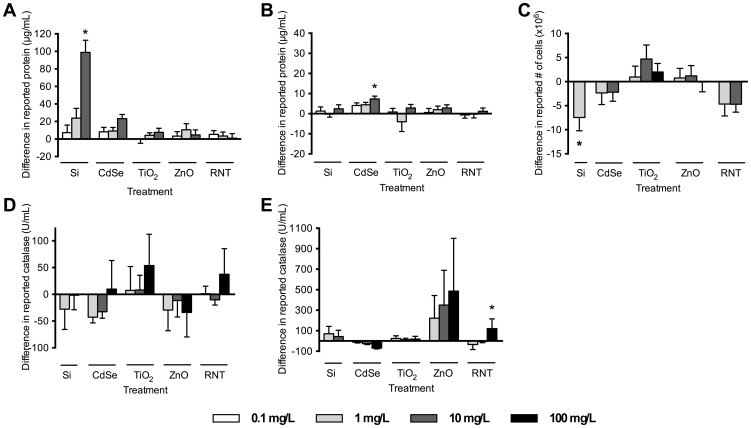
Assessment of nanoparticle interference with the assays components, with addition of analyte. (A) BCA protein assay with 250 µg/mL BSA addition; (B) Bradford protein assay with 40 µg/mL BSA addition; (C) LDH assay with 12.5×10^6^ lysed cells; (D) Catalase assay with 250 U/mL catalase addition (excitation 531 nm, emission 595 nm); (E) Catalase assay with 250 U/mL catalase addition (absorbance 560 nm). * indicates significantly different than control (p<0.05, ANOVA followed by Dunnett's post-hoc comparison).

The optical properties of a NP can interfere with the endpoint measurement of absorbance or fluorescence in a biochemical assay. For example, the absorbance spectrum of gold NPs overlaps with the absorbance range measured in a hemolysis assay, leading to erroneous results [26]. Possible interference effects (such as surface plasmon resonance and quantum confinement) that can originate from varying size, shape, composition, surface modality and inter-particle interaction [27], [28] make optical characterization of each NP species essential. Of the tested NPs, Si were the only ones to absorb (0.28 a.u. at 340 nm, Figure 2). While Si NPs affected the BCA assay (Figure 3), they only absorbed 0.052 a.u. at 562 nm (Figure 2), therefore this was likely not the source of interference in this case. Si NPs did interfere with the LDH assay which is measured at 340 nm (Figure 4C); however, this assay is based on the rate of absorbance change as opposed to final absolute absorbance, therefore in this case the effect of the absorbance of the NPs is negated. It is likely that in this case interference is the result of direct interaction between Si and the enzyme [6] as discussed below, rather than via the intrinsic absorbance of the Si. The other NPs used in our study did not fluoresce or absorb at wavelengths monitored in the presented assays but they still affected the results, indicating other sources of assay interference.

### Spectroscopic assays

Many spectroscopic protocols may not be appropriate for use in NP toxicity testing; every assay in the current study was affected by at least one NP formulation and each NP formulation tested, with the exception of ZnO, affected at least one assay (Figure 3, 4). There does not appear to be any obvious link between measured NP physicochemical properties and the observed interference with spectroscopic assays tested within.

#### Interference of nanoparticles with assay components without analyte

Solely adding NPs to the assay components and performing the assay as suggested by the manufacturer can lead to false positive results. In many cases a substantial underestimation of toxicity occurs in the presence of NPs, even without any analyte. Both the MTS and alamar blue assays were affected by exposure to NPs (Figure 3C, D). CdSe 100 and RNT 100 interfered with the MTS assay, calculating the equivalent of 547±224 and 1118±89 cells respectively (Figure 3C), and CdSe 100 and TiO_2_ 100 affected the alamar blue assay (Figure 3D), measuring the equivalent of 1297±50 and 593±173 cells respectively, despite no presence of cells in the assay. These assays are often used as proxies of cytotoxicity, viability, or proliferation *via* cell counts; this type of data artefact could lead researchers to overlook a toxic NP effect. Reports of protein concentration, as in the BCA assay are often used to normalize values of enzyme activity and inaccurately high values can lead to an underestimation of damage. At relatively low concentrations, Si 1 (12±2 µg/mL), Si 10 (98±6 µg/mL) and CdSe 10 (13±2 µg/mL) interfere with the BCA protein assay (Figure 3A). An artefact in this assay would further exacerbate the problem if was used in conjunction with other assays that are similarly affected by the presence of NPs. In other cases, we found that the presence of NPs in assays commonly used to test for oxidative stress caused effects. The activity of catalase in the presence of TiO_2_ was inaccurately reported in both the fluorescence-measured assay (120±44 U/mL) and the absorbance-measured assay (110±22 U/mL) (Figure 3E, F). This finding is of substantial significance as oxidative stress is often reported as the main cause of *in vitro* toxicity associated with NP exposure [29]. This leads to critical difficulties in differentiating between true oxidative stress and artefacts caused by NP-assay interference. It is clear from these data that NPs have the potential to interfere with the components of the assays themselves; however, the use of these assays clearly requires the addition of a sample for measurement. Therefore, we assessed the validity of some of the assays under more realistic test conditions by addition of biological samples (analytes).

#### Interference of nanoparticles with assay components with addition of analyte

Identifying NP interference with assay reagents alone cannot always be used to predict interference under the final working conditions of the assay. We found that in some cases, addition of a protein (*i.e.* BSA in the protein assays and catalase in the catalase activity assay), could either eliminate or enhance the interference observed when only assay reagents and NPs were mixed (Figure 4). The interference caused by Si 1 and CdSe 10 in the BCA assay (Figure 3A) is abolished by the addition of BSA (Figure 4A), and similarly, TiO_2_ 100 effects in the catalase assay (Figure 3E, F) are abrogated in the presence of catalase (Figure 4D, E). Therefore, the use of these assays may be acceptable under these more realistic conditions. However, of concern is the incidence of erroneous results with the addition of analyte. While NPs did not affect the Bradford assay with no protein present (Figure 3B), addition of 40 µg/mL of BSA with CdSe 10 over-estimated by 7±1 µg/mL BSA concentration (Figure 4B). Similarly, RNT did not affect the catalase activity assay components themselves (Figure 3F), but in the presence of catalase, an overestimation of 120±96 U/mL is observed (Figure 4E). The only case where NP interference with assay components alone was an accurate indicator of compatibility with the assay during practical use was the addition of Si 10 in the BCA assay (Figure 3A, 4A); NP incubated with assay components yielded a value of 98±6 µg/mL, and with the addition of BSA this value was 99±14 µg/mL. This suggests that simply performing controls with assay components and NPs alone may not be enough to confirm whether an assay is appropriate for use with NPs. Furthermore, there are many assays where performing a ‘control’ by the addition of analyte is not possible due to the complex nature of the assay itself. Many assays used for cytotoxicity testing (e.g. MTS and alamar blue) rely on live cells to metabolically convert dyes. It is experimentally difficult to reproduce these situations in order to perform accurate controls. In the case of the LDH cytotoxicity assay, we could perform an analogous control by lysing cells to measure LDH activity (Figure 4C). We found that the presence of 1 mg/L Si resulted in a reported number of 7.4×10^6^ cells, which was significantly lower than the actual 12.5×10^6^ cells present (Figure 4C). In an experimental situation, this would have resulted in significant overestimates of cell death in NP-treated cells. Other studies suggest that this assay is susceptible to a number of different NPs (*e.g.* CdSe [6]; soot, carbon, TiO_2_ [4]). However, we found that neither CdSe nor TiO_2_ affected the assay in this instance. This may be attributed to differences in NP physicochemical characteristics between studies as well as our use of lysed cell preparations instead of purified LDH. Lysed cell preparations will contain a myriad of proteins and macromolecules that may bind the NPs and prevent interactions with LDH, a mechanism that will not be present in purified LDH samples.

#### Nanoparticle and molecule interactions

NPs readily bind to various macromolecules and such interactions have been exploited in applications such as environmental remediation, imaging, and detection. Our data suggests that these interactions may also affect the outcome of spectrophotometric assays. Differences in the nature and magnitude of NP-assay interference with and without protein present suggests strong interactions are occurring between NPs and proteins in the assay [30]. These interactions appear to alter the effects of NPs on assays and may further complicate the task of predicting and controlling for potential interference. NPs can change the conformation of proteins and decrease or even stabilize enzyme activity under denaturing conditions [6], [31]. In addition, the presence of proteins can affect the stability and agglomeration state of the NPs themselves [32], which may influence the characteristics of NP exposure. LDH assays have been shown to be affected by NPs through the interaction of NPs with the LDH enzyme itself, causing adsorption and/or inactivation of the protein and an associated loss of activity [4], [6], [15]. Circular dichroism spectroscopy revealed that 100 mg/L Si binds to LDH and changes its native structure, subsequently altering the activity of the enzyme [6]. However, this interaction is abolished with the addition of BSA, which likely binds the NPs and reduces associations with LDH [6]. CNTs bind to phenol red, but in the presence of serum this association decreases significantly [33]. In the current study, addition of protein and enzyme to the BCA and catalase assays may have decreased the availability of NPs to catalyze reduction of the dyes. However, in the case of the Bradford assay, none of the NPs tested affected the Bradford protein assay in the absence of protein (Figure 3B), but when 40 µg/mL BSA was added, the CdSe treatment falsely reported 46±1 µg/mL protein (Figure 4B). The CdSe were highly agglomerated (Table 1), and addition of protein may have led to dispersal and stabilization of the NPs in suspension, which could have increased NP binding to assay components [6], [34].

Electrostatic interactions between NPs and the components of the assays and biological samples are likely to occur [35], [36]. If electrostatic interactions were the main predictor of interference, we would expect to observe differences in interference between positively and negatively charged NPs. In our experiments, using positively charged RNTs resulted in a significantly erroneous over-quantification of 1118±89 cells (Figure 3C), despite an absence of cells in the assay. This is consistent with a previously published study that reported that RNTs can affect the final measurement of the negatively charged tetrazolium dye [37]. However, when negatively charged CdSe were added to the MTS assay, a significant over-estimation of 547±224 cells was also observed (Figure 3C). Further, RNTs do not always affect negatively charged dyes, as no interference was apparent with resorufin in the alamar blue assay (Figure 3D). These data suggest that interference cannot be easily predicted solely using the basic characteristics of the NPs [4].

#### Nanoparticle-related dye reduction

Interestingly, we observed an increase in final absorbance or fluorescence in almost every assay when NPs were present. This suggests that NPs are interacting with the final form of the dyes in such a way as to enhance their absorbance or fluorescence, and/or that the NPs themselves are causing reduction of the dyes, leading to a higher concentration of the final form of the dye. In many studies, quenching of fluorescent dyes is observed for alamar blue [19] and DCF dye, the final product of the oxidative stress marker dichlorodihydrofluorescein diacetate (H_2_DCF-DA) [5]. However, in our study, incubation of CdSe 100 or TiO_2_ 100 with alamar blue reagent resulted in much higher fluorescence than expected in the current study, reporting 1297±50 cells and 593±173 cells, respectively (Figure 3D), and was also observed with TiO_2_ in the catalase assay (Figure 3E, F). Similar observations of DCFH-DA dye fluorescence enhancement in the presence of gold or iron oxide NPs have been observed [38], [39]. Free electron transfer from excited CdSe or TiO_2_ semiconductor NPs may contribute to this phenomenon [40]. Many assays are dependent on redox reactions to generate a colorimetric or fluorometric signal, and the small size of metal NPs can enhance the reduction potential of these materials, which may allow NPs to reduce dyes in the absence of cellular activity [21], [41]. For example, the BCA protein assay is dependent on the reduction of Cu^2+^ to Cu^+^ by a protein. In the absence of protein, we hypothesize that NPs may drive this reduction, causing Cu^+^-mediated dye interaction and resulting in erroneous protein concentration measurements. Metal NPs can catalyze redox reactions [42], [43], and this may account for some of the results presented here. Regardless of the mechanism at work, it is clear that NPs impact all of the assays investigated here and care must be exercised when using these methods for nanotoxicity testing.

### Literature survey

**Figure 5:**
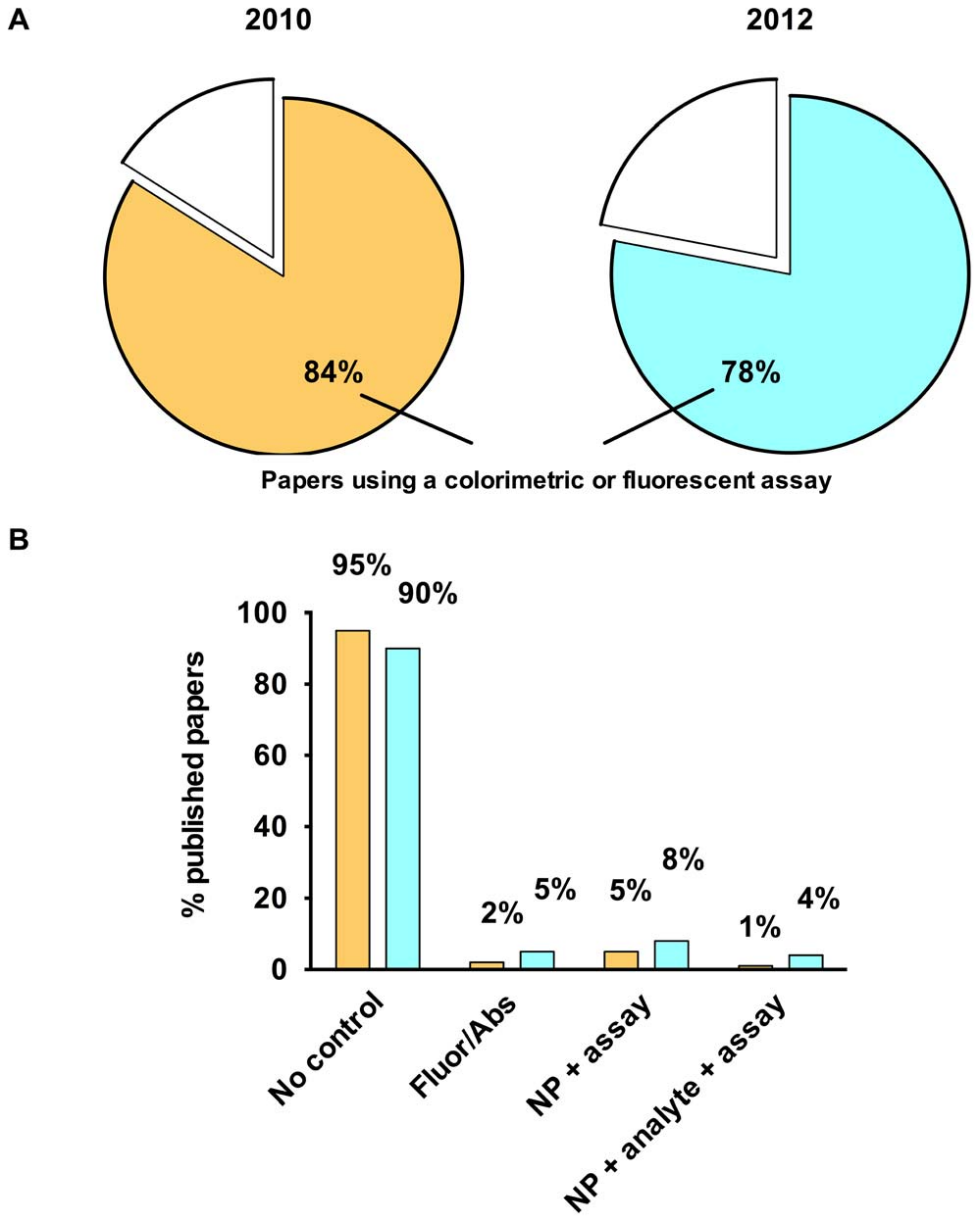
Literature survey to determine the percentage of papers testing for nanoparticle interference in spectroscopic-based assays. (A) Percentage of published papers that use a toxicity assay based on measurement of colorimetric or fluorescent change in either 2010 or 2012; (B) Breakdown of controls performed in papers using one of these assays. (note: Percentages do not add up to 100% due to overlap in papers performing more than one control).

Clearly, toxicological/biological studies require controls to validate whether a particular assay is appropriate for each NP formulation. Our analysis of the literature from 2010 demonstrates that ∼84% of papers in the nanotoxicology field used at least one type of colorimetric or fluorescence assay (Figure 5A) and of these, ∼95% were published without reporting controls for NP interference (Figure 5B). Even with an ever increasing number of published reports on NP-assay interference, this number has only marginally improved; a re-analysis of the literature from 2012 shows that ∼90% of papers were published without some type of assay control (Figure 5B). Reporting in all areas monitored in this study was slightly improved in 2012 (Figure 5B). The most common control performed was the addition of NPs to the assay components alone (2010: 5%, 2012: 8%), followed by measurement of the intrinsic fluorescence or absorbance of the NPs (2010: 2%, 2012: 5%), then with NPs and an analyte (2010: 1%, 2012: 4%) (Figure 5B). The results of this study highlight the critical need for more stringent requirements for the use of these types of assays in nanotoxicity testing. The misinformation resulting from NP-assay interference has substantial implications for our understanding of, and confidence in the reported bioactivity of NPs. We believe that such problems have contributed significantly to the conflicting reports of NP toxicity in the literature.

### Recommendations for future nanotoxicology studies

We suggest that more stringent controls be required for nanotoxicological studies and provide the following recommendations to minimize the potential for NP-assay interactions and associated aberrant results. Higher concentrations of NPs (>10 mg/L) have greater probability of interfering with assay function, and the use of such concentrations is not uncommon in toxicological studies. Therefore, NP concentration should be limited in the final sample, recognizing that even with multiple washes and/or centrifugations NPs could remain within cells or bound to membranes [1], [44]. Furthermore, centrifugation can be counterproductive if NPs have bound to the assay components, inadvertently removing dyes and/or proteins essential for accurate readings [4]. Researchers should note that final NP concentration present in the assay will likely already be lower than experimental nominal concentration due to factors such as incomplete membrane translocation, or binding to mucus or serum components [45]. We show that the addition of analytes (*i.e.* cells, tissue sample, *etc.*) will modify the degree of NP interference, therefore the practice of testing for interference by measuring addition of NP to assay reagents may not be adequate. If possible, assays should be tested with the analyte in question to determine if interactions will occur with the assay components. Some assays may be deemed unreliable for nanotoxicity assessment [46]. Given that we are currently unable to accurately predict how each NP will interact, it is imperative that each individual formulation be tested for compatibility with all assays used. Such quality control practices will allow for the appropriate interpretation and evaluation of published results and provide accurate scientific data for the establishment of regulations related to safe NP production, utilization, and disposal.
